# Comparison of non-invasive diagnostic modalities for ocular surface squamous neoplasia at a tertiary hospital, South Africa

**DOI:** 10.1038/s41433-023-02833-0

**Published:** 2023-11-23

**Authors:** Roland Hӧllhumer, Pamela Michelow, Susan Williams

**Affiliations:** 1 Department of Neurosciences, Division of Ophthalmology, University of the Witwatersrand, Johannesburg, South Africa; 2 Cytology Unit, National Health Laboratory Service and Department of Anatomical Pathology, Faculty of Health Sciences, University of the Witwatersrand, Johannesburg, South Africa

**Keywords:** Eye cancer, Diagnosis

## Abstract

**Aims:**

The aim of the study is to assess non-invasive diagnostic modalities for ocular surface squamous neoplasia (OSSN) when compared to histology.

**Methods:**

A prospective case–control study was conducted of patients presenting with conjunctival masses at a tertiary eye hospital in Johannesburg, South Africa. Patients completed an interview and had three non-invasive diagnostic tests: optical coherence tomography, impression cytology and methylene blue stain. A biopsy with histology was performed as the gold standard to confirm the diagnosis.

**Results:**

One hundred and eighty-two conjunctival masses of 175 patients were evaluated. There were 135 lesions identified as OSSN on biopsy and 47 lesions were benign on histology. Optical coherence tomography had a sensitivity and specificity of 87.2% (95% CI: 80.0–92.5) and 75.6% (95% CI: 60.5–87.1), respectively, when an epithelial thickness cutoff of 140 um was used. Shadowing was found in 46% of cases due to leukoplakia or increased thickness of the mass. Cytology had a sensitivity of 72.4% (95% CI: 62.5–81.0) and a specificity of 74.3% (95% CI: 56.7–87.5). Twenty-seven per cent of cytology specimens were excluded from analysis due to inadequate cellularity. Methylene blue had a high sensitivity of 91.9% (95% CI: 85.9–95.9), but low specificity of 55.3% (95% CI: 40.1–69.8).

**Conclusion:**

Optical coherence tomography had a high sensitivity and specificity as a non-invasive test and liquid-based cytology performed well but had a lower sensitivity and specificity than with optical coherence tomography. Methylene blue performed well as a screening test, with a high sensitivity but low specificity.

## Introduction

Ocular surface squamous neoplasia (OSSN) is the most common ocular surface tumour and includes premalignant conjunctival intraepithelial neoplasia (CIN), malignant squamous cell carcinoma in situ (CiS) and invasive squamous cell carcinoma (SCC). Incidence rates range from 0.03 to 3.4 per 100,000 persons/year, with significant geographic variability [1].

OSSN is suspected clinically with the presence of an elevated pearly grey conjunctival lesion with a variable amount of pigmentation, leukoplakia and feeder vessels or the presence of a diffuse pearly lesion extending from the conjunctiva onto the cornea [2]. The gold standard for diagnosis is a biopsy with histology [3]. OSSN is staged according to the American Joint Committee on Cancer Staging criteria of 2017 (Supplement 1) [4]. In recent years, there has been a move from surgical management to the use of topical chemo or immunotherapies [5]. This has spurred the adoption of non-invasive modalities for diagnosis and the monitoring of response to therapy. These diagnostic methods include optical coherence tomography (OCT), cytology, vital dye stains (methylene blue and toluidine blue) and confocal microscopy [3].

OCT has emerged as a useful tool to acquire an optical biopsy of a conjunctival lesion [6–9]. The classic features of OSSN on OCT are a (i) thickened hyperreflective epithelium, (ii) an abrupt transition from normal to abnormal epithelium, (iii) and a plane of separation between the epithelium and underlying stroma [6]. This plane of separation may be absent in invasive disease and can be masked in lesions that have leukoplakia or in masses with increased thickness [6, 7].

Cytology has been used widely for the diagnosis of cervical cancers, but its use in the diagnosis of OSSN is less common [10, 11]. Specimens for conjunctival cytology can be collected by exfoliation of the mass or by impression with a nitrocellulose filter [12]. These can be transported to the laboratory in a traditional alcohol-based medium or newer liquid-based cytology medium. Liquid-based cytology uses a proprietary transport medium and automated preparation process to simplify assessment [13]. It additionally allows for the storage of cellular material for use in ancillary investigations such as polymerase chain reaction (PCR) [13]. This is relevant in OSSN as human papilloma virus has been thought to be associated with OSSN [1].

Vital dyes, such as methylene and toluidine blue, stain cancer cells and have therefore been described for the diagnosis of OSSN [14–16]. The cancer cells stain due to their affinity for nucleic acids and their accumulation in intercellular spaces [14–17]. They have been shown to have a high sensitivity but low specificity for OSSN, which has limited their use in clinical practice [14–16].

Our study compared three non-invasive diagnostic modalities (OCT, cytology, methylene blue) to the gold standard of histology for the diagnosis of OSSN, in an urban South African hospital population.

## Materials and methods

This prospective case–control study was conducted at a tertiary eye hospital in Johannesburg, South Africa. Ethics approval was granted by the Human Research Ethics Committee of the University of the Witwatersrand (M190729) and adhered to the tenets of the Declaration of Helsinki. The clinical is registered with the Pan African Clinical Trials Registry (PACTR201912900667480).

The study included patients who presented with conjunctival masses between December 2019 and February 2022. Recruitment was not consecutive, as this period coincided with the COVID-19 pandemic. Patients were recruited if they had conjunctival masses that were either considered to be suspicious for OSSN or were considered benign but remained symptomatic despite medical therapy (topical lubricants and/or corticosteroids). Features of OSSN included a raised lesion with feeder vessels, a mass with leukoplakia, pigmented mass, and a diffuse pearly lesion extending from the conjunctiva onto the cornea. Exclusion criteria for enrolment included age less than 18 years; females that were pregnant or breastfeeding; a history of previous topical chemotherapy or surgery in the involved eye; lesions with a basal diameter of greater than 15 mm or where adjacent structures other than the cornea or sclera were involved; conditions that prevented performing study investigations; the presence of conditions that are known to predispose to OSSN (xeroderma pigmentosum); or a diagnosis of primary acquired melanosis.

Patients who met the inclusion criteria underwent informed consent and completed an interview to document demographic data, history of presenting complaint and the presence of associated risk factors (HIV status, UV exposure history, smoking, immunosuppressive conditions or medication, ocular surface inflammation, ocular injury, regular exposure to petroleum products). A clinical examination and anterior segment photography were performed with slit lamp to document clinical features. OCT and methylene blue stain were performed at the initial visit, with impression cytology and biopsy for histology at the time of surgery.

**Fig. 1:**
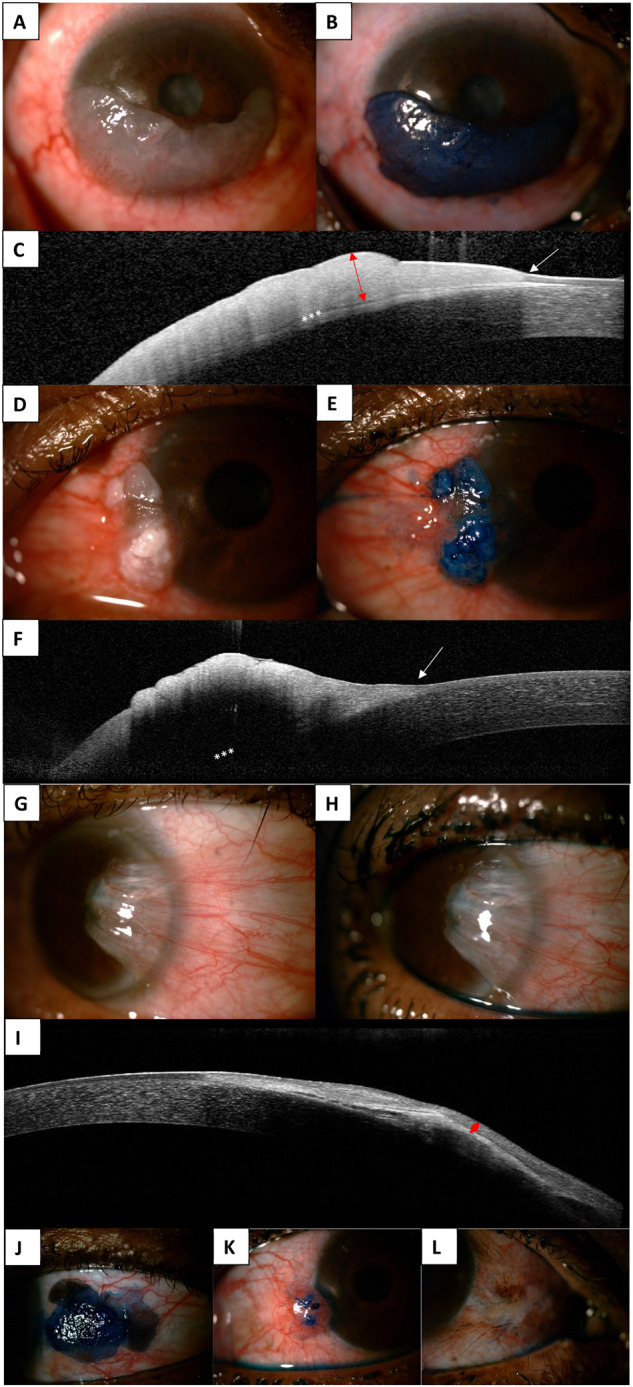
Imaging of ocular surface squamous neoplasia. **A** Anterior segment photo of a diffuse gelatinous OSSN that has **B** a diffuse stain with methylene blue and **C** shows a thickened hyperreflective epithelium (red arrow) with transition zone (white arrow) and plane of separation (white asterisk). **D** Anterior segment photo of a leukoplakic OSSN that has **E** a diffuse stain with methylene blue and **F** a thickened hyperreflective epithelium on OCT with transition zone (white arrow), but masking of the underlying plane of separation (white asterisk). **G** Anterior segment photo of a pterygium with **H** no stain on methylene blue and **I** a normal epithelium (red diamond). **J**–**K** Methylene blue staining of OSSN, highlighting **J** a diffuse, **K** focal and **L** speckled pattern of staining.

An anterior segment OCT was performed using spectral domain OCT with an 880 nm infrared light source (Heidelberg Engineering, Heidelberg, Germany). A large corneal scan with 21 sections and a 20-degree arc was performed. Sections were reviewed by the PI (RH) to document the maximum epithelial thickness, the presence of a transition zone, plane of separation, and shadowing (Fig. 1). The reviewer was blinded to the histology and cytology results when the scans were assessed. Epithelial thickness was measured manually with a digital calliper. If shadowing was present or the epithelial plane was not visible in large lesions, no epithelial thickness was recorded. Even though maximum epithelial thickness could not be measured in all cases, the epithelium could still be assessed for a thickened hyperreflective epithelium, transition zone and plane of separation where shadowing was not present.

Methylene blue staining was performed after administering a topical anaesthetic drop. A single drop of 1% methylene blue was instilled into the inferior fornix, the eye was lightly closed for 30 s after which the dye was rinsed out with sterile water drops, and an anterior segment image was taken to document the staining pattern. The lesion was documented as staining if there was complete or partial uptake of the stain by the mass. The staining pattern was described as diffuse, focal or speckled. A diffuse pattern was where the entire lesion took up stain, focal when there was patchy uptake of the stain and speckled when there was a speckled pattern of uptake (Fig. 1).

Impression cytology was performed at the start of the surgery. Three sequentially applied filter papers with a pore size of 0.45 um (Merck, Millipore, Cork, Ireland) were applied with the aim of increasing the cellularity of specimens. The filter papers were applied with light pressure over the lesion for 10 s, placed in a vial containing ThinPrep preservation solution (Hologic, Massachusetts) and sent to the laboratory. Once in the laboratory, the specimens were processed using the ThinPrep 2000 processor (Hologic, Massachusetts). Cytology specimens were then examined under the microscope and reported using the same criteria as the 2014 Bethesda System for reporting cervical cytology, the categories being negative for intraepithelial lesion or malignancy (NILM), atypical squamous cell of unknown significance (ASC-US), low-grade squamous epithelial lesion (LSIL), high-grade squamous intraepithelial lesion (HSIL) and invasive SCC [18]. All cytology specimens were examined by one cytologist (PM) who was blinded to the histology results. For the purpose of this study ASC-US, LSIL, HSIL and SCC were considered as a positive cytological diagnosis for OSSN. Only specimens with good cellularity were included for analysis.

All patients had a biopsy to confirm the diagnosis by histology. Histology was used as the gold standard for comparison with the other diagnostic modalities (OCT, cytology, methylene blue). Histology was performed with the support of a clinical history and not by a dedicated member in the research team. The pathologists were blinded to the other investigations. Masses suspicious of OSSN that occupied less than or equal to four clock hours of the limbus had an excision biopsy with 4 mm margins using the Shields no-touch technique and cryotherapy. Keratoepitheliectomy was performed if there was extension onto the corneal surface and partial sclerectomy if there was scleral invasion. Larger lesions had an incision biopsy performed and received topical chemotherapy once the epithelium had healed. Lesions that were clinically considered benign had a simple excision and conjunctival autograft. Excision biopsies were mounted on a sponge with orientation sutures. Biopsies were formalin fixed, paraffin embedded and sectioned. They were then stained with a haematoxylin and eosin stain and examined under the microscope. Frozen section was not undertaken. No histology specimens were inadequate for analysis.

Data analysis was performed using STATA (StataCorp LLC, Texas, USA), version 17.0. A total sample size had been calculated at *n* = 173, for a sensitivity of 90%, to detect a difference of 10% with a 95% confidence interval. Descriptive statistics were used for patient characteristics, clinical features, and associated risk factors. Shapiro–Wilk test was used to test for normality on continuous variables. Categorical data are presented as numbers and percentages. Continuous data that do not show a normal distribution are summarised with medians and interquartile range. Wilcoxon rank-sum test was used to compare continuous variables that do not have a normal distribution. The *χ*^2^ or Fisher’s exact test was used to compare categorical variables. A receiver operating characteristic (ROC) curve was used to determine the epithelial thickness cutoff for OCT analysis. A significance level of *p* < 0.05 was used.

## Results

One hundred and eighty-two conjunctival masses of 175 patients were included in this study. One hundred and thirty-five (74%) of the conjunctival masses (in 130 patients) were identified as OSSN on histology and 47 (26%, in 45 patients) were benign on histology. The detailed baseline characteristics of the participants and the histology results are summarised in Supplement 2.

**Fig. 2:**
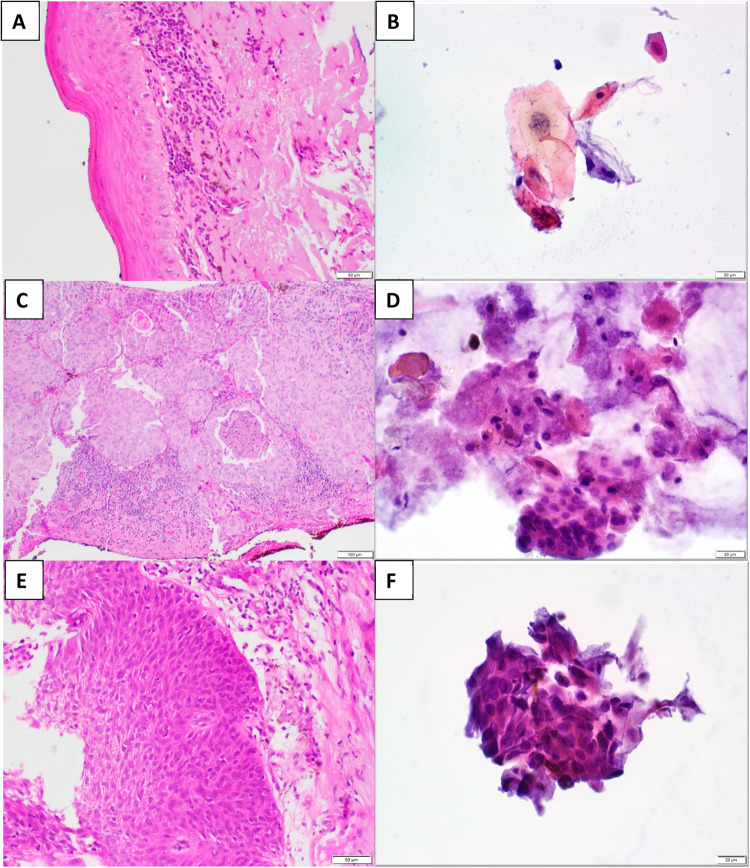
Histology and cytology of ocular surface squamous neoplasia. **A** Histology showing preserved polarity in basal layers with large and hyperchromatic nuclei in superficial layers consistent with a low-grade squamous intraepithelial lesion (H+E ×40). **B** Cytology comprising atypical cells with abundant cytoplasm and enlarged irregular nuclei consistent with a low-grade squamous intraepithelial lesion (Papanicolaou stain ×40). **C** Large malignant squamous cells, seen on histology, with eosinophilic cytoplasm, intercellular bridges and enlarged nuclei invading into underlying stroma consistent with squamous carcinoma (H+E ×20). **D** Malignant squamous cells in a necrotic background are consistent with squamous carcinoma on cytology (Papanicolaou stain ×40). **E** Thickened epithelium with loss of maturation and full thickness dysplasia consistent with high-grade squamous intraepithelial lesion on histology (H+E ×20). **F** A fragment of tissue comprising cells with a high nuclear: cytoplasmic ratio and hyperchromatic nuclei with irregular nuclear contours on cytology (Papanicolaou stain ×40). Histology slides courtesy of Dr Van De Byl, Chris Hani Baragwanath Hospital Anatomical Pathology, University of the Witwatersrand and National Health Laboratory Service.

Figure 1 shows representative images of participants with OSSN that were investigated with methylene blue, OCT, cytology and histology. Figure 2 describes the cytology and histology features of the different grades of OSSN on cytology.

Table 1 gives a detailed breakdown of the detection rate for OSSN of the various non-invasive tests, stratified according to the histology results.

**Table 1:** Breakdown of the yield of the non-invasive diagnostic tests.

	Histology	Non-invasive tests	
		Positive for OSSN	Negative for OSSN
OCT	170	120	50
Benign	45	11	34
CIN	107	91	16
CiS	5	5	0
SCC	13	13	0
Cytology	133	98	35
Benign	35	9	26
CIN	81	56	25
CiS	4	4	0
SCC	13	11	2
Cytology in OSSN with leukoplakia	55	36	13
Cytology in OSSN without leukoplakia	78	35	14
Methylene Blue	182	145	37
Benign	47	21	26
CIN	112	101	11
CiS	6	6	0
SCC	17	17	0

*OCT* optical coherence tomography, *CIN* conjunctival intraepithelial neoplasia, *CiS* squamous cell carcinoma in situ, *SCC* squamous cell carcinoma, *OSSN* ocular surface squamous neoplasia.

Table 2 outlines the diagnostic accuracies of the different modalities when compared to histology as the gold standard. OCT performed the best overall with a sensitivity of 87.2% (95% CI: 80.0–92.5) and specificity of 75.6% (95% CI: 60.5–87.1). Impression cytology performed well with a sensitivity and specificity of 72.4 (95% CI: 62.5–81.0) and 74.3% (95% CI: 56.7–87.5). Combining tests did not yield better results (Supplement 3). Combining Methylene blue and cytology gave the highest sensitivity at 94.8% (95% CI: 89.6–97.9), but at the expense of specificity (46.8%, 95% CI: 32.1–61.9). This is largely due to methylene blue that has a high sensitivity but low specificity. When using methylene as a screening test, followed by OCT or cytology of those cases that had staining, the sensitivity of cytology and OCT improved, but again at the expense of specificity.

**Table 2:** Comparison of the diagnostic modalities of cytology, OCT and methylene stain for OSSN when compared to the histological diagnosis.

	Sensitivity	Specificity	PPV	NPV	+ Likelihood ratio	– Likelihood ratio
OCT	87.2 (80.0–92.5)	75.6 (60.5–87.1)	90.8 (84.2–95.3)	68.0 (53.3–80.5)	3.57 (2.12–5.99)	0.17 (0.10–0.28)
Cytology	72.4 (62.5–81.0)	74.3	88.8 (79.7–94.7)	49.1 (35.1–63.2)	2.82 (1.58–5.01)	0.37 (0.25–0.54)
Cytology in OSSN with leukoplakia	73.5 (58.9–85.1)	33.3 (4.3–77.7)	90.0 (76.3–97.2)	13.3 (1.7–40.5)	1.10 (0.61–1.99)	0.80 (0.23–2.71)
Cytology in OSSN without leukoplakia	71.4 (56.7–83.4)	82.8 (64.2–94.2)	87.5 (73.2–95.8)	63.2 (46.0–78.2)	4.14 (1.83–9.38)	0.35 (0.22–0.55)
Methylene	91.9 (85.9–95.9)	55.3 (40.1–69.8)	85.5 (78.7–90.8)	70.3 (53.0–84.1)	2.06 (1.49–2.84)	0.15 (0.08–0.27)

*OSSN* ocular surface squamous neoplasia, *OCT* optical coherence tomography, *PPV* positive predictive value, *NPV* negative predicative value.

One hundred and seventy participants had an OCT. A ROC curve was used to determine the cutoff value for epithelial thickness. A value of 140 um was selected as the optimal cutoff point (Supplement 4). The discrimination in detecting OSSN on OCT was 0.81 (95% CI: 0.74–0.87) (Supplement 5). Shadowing was present in 79 patients, either due to the presence of leukoplakia or due to the thickness of the mass. The mean epithelial thickness in the OSSN masses was 295 um (95% CI: 71–730), while the mean thickness in benign masses was 98 um (95% CI: 52–236). Epithelial thickness could reliably be measured up to a thickness of 457 um. Sensitivity and specificity improved with stage of disease, where no patients with SCC had a missed diagnosis (Table 3).

**Table 3:** Diagnostic accuracy of OCT, cytology and methylene blue when compared to histology for different stages of disease.

	Sensitivity	Specificity	PPV	NPV	+ Likelihood ratio	– Likelihood ratio
OCT						
CIN	85.0 (76.9–91.2)	54.0 (40.9–66.6)	75.8 (67.2–83.2)	68.0 (53.3–80.5)	1.85 (1.40–2.44)	0.28 (0.17–0.46)
SCC	100 (75.3–100)	31.8 (24.6–39.7)	10.8 (5.9–17.8)	100 (92.9–100)	1.47 (1.32–1.63)	Too few cases
Cytology						
CIN	69.1 (57.9–78.9)	53.8 (39.5–67.8)	70.0 (58.7–79.7)	52.8 (38.6–66.7)	1.50 (1.08–2.08)	0.57 (0.38–0.87)
SCC	84.6 (54.6–98.1)	42.5 (33.5–51.9)	13.8 (7.1–23.3)	96.2 (87.0–99.5)	1.47 (1.11–1.94)	0.36 (0.10–1.32)
Methylene						
CIN	90.2 (83.1–95.0)	37.1 (25.9–49.5)	69.7 (61.5–77.0)	70.3 (53.0–84.1)	1.43 (1.19–1.74)	0.26 (0.14–0.50)
SCC	100 (80.5–100)	22.4 (16.3–29.6)	11.7 (7.0–18.1)	100 (90.5–100)	1.29 (1.19–1.40)	Too few cases

*PPV* positive predictive value, *NPV* negative predictive value, *OCT* optical coherence tomography, *CIN* conjunctival intraepithelial neoplasia, *SCC* squamous cell carcinoma.

One hundred and eighty-two specimens were submitted for cytology. Forty-nine specimens (27%) did not have adequate quality for analysis, 37 (27%) of the OSSN masses and 12 (26%) of the benign masses. Forty-five per cent (*n* = 22) of the specimens that were inadequate for analysis had leukoplakia, whereas 41% (*n* = 55) of the representative specimens had leukoplakia. The yield from cytology improved with increasing stage of disease, with a sensitivity of 84.6% (95% CI: 54.6–98.1) in SCC (Table 3). The discrimination in detecting OSSN on cytology was 0.73 (95% CI: 0.65–0.81) (Supplement 5). In the first half of the study (*n* = 66), this was 0.76 (95% CI: 0.64–0.87) and in the last half of the study it was 0.71 (0.58–0.84). The presence of leukoplakia had a negative effect on the specificity of cytology, with this being reduced from 82.8 (95% CI: 64.2–94.2) to 33.3% (95% CI: 4.3–77.7) in the presence of leukoplakia (Table 2).

Methylene blue was performed in all patients and performed well as a screening test with a sensitivity of 91.9% (95% CI: 85.9–95.9) (Table 2). The discrimination in detecting OSSN on cytology was 0.74 (95% CI: 0.67–0.8) (Supplement 5). There was no improvement in results when analysing methylene blue according to different staining patterns.

## Discussion

OSSN is the most common ocular surface tumour and has traditionally been diagnosed by histology. With the increased uptake of topical therapy as the primary therapeutic modality, there has been an increased adoption of non-invasive modalities for the diagnosis of OSSN and the monitoring of response to therapy. Our study reports on three diagnostic methods: OCT, cytology and methylene blue staining, when compared to histology as the gold standard for diagnosis.

OCT has become the favoured non-invasive diagnostic modality for OSSN [3]. Studies have reported sensitivities of 94–100% with specificities of 100% [6, 7]. Our study had lower sensitivity and specificity overall, but with a 100% sensitivity for SCC. The OCT used in this study was a commercial high-resolution OCT, whereas many earlier studies used a non-commercial ultra-high-resolution OCT [6]. This may have contributed to the lower sensitivity and specificity in our study. Central to the diagnosis of OSSN is the presence of a thickened hyperreflective epithelium. The literature does not have a consensus on what value should be used for a thickened epithelium. Looking at the ROC curve, we used 140 um as the cutoff in our study. Published studies have used values that range 120–142 um, all with good results [6, 7]. Our study had a large number of patients with shadowing of the plane of separation (46%), either due to leukoplakia or an increased thickness of the mass. Although this affects the ability to measure and report on the area of the thickest epithelium, one is still able to assess the scan for the three diagnostic features on OCT when reviewing the edge of the mass. Nanji et al. [7] found shadowing in 24% of their cases, with these lesions mostly thicker than 465um. We had similar results with reliable thickness being measured up to 457 um. Our study confirms that OCT is a useful diagnostic modality for OSSN.

Cytology was first described in 1997, but there remains a paucity of reports in the literature [10]. Considered a non-invasive procedure, it has the benefit over the other two modalities in that it looks at the pathology at a cellular level. Despite this, it has not yielded results superior to OCT. In our study, we had sensitivity and specificity of 72.4 and 74.3%, respectively. A small case series reported a sensitivity and specificity of 92 and 95% for detecting CIN, where pterygia were controls [19]. They used a different technique for processing, with the impression filters being fixed immediately. Our study is the first to report on the use of liquid-based cytology in OSSN. This places the filter papers with the cellular material into a liquid medium that undergoes automated processing before analysis. Twenty-seven per cent of our specimens did not have adequate cellularity for processing and so it is possible that the volume of cells removed from the ocular surface is not optimal for this method of processing. A future study could compare the cellularity and diagnostic accuracy of fixation versus liquid-based cytology. Previous studies have reported a reduced yield of cytology in patients with hyperkeratosis [11, 19, 20]. We did not find a significant difference in number of cases with leukoplakia between representative and non-representative specimens (41 vs. 45%).

Methylene blue is a vital dye that stains cancer cells [15]. This can be used in the diagnosis of cancers, but also for the delineation of tumours at surgery, to ensure they are removed with adequate margins [15, 16]. One study from South Africa reported a sensitivity of 97% and specificity of 50% when using methylene blue to stain conjunctival masses. They however grouped benign and premalignant (CIN1 and 2) into one group and SCC or CIN3 in the second. We followed a more traditional classification of OSSN vs. benign lesions and found a similar sensitivity of 92% and specificity of 55%. It is therefore a good test for the exclusion of OSSN and might be well placed in the primary care setting for patients presenting with conjunctival masses, to determine urgency of referral. We considered using this as a screening test before either cytology or OCT. This did not however improve diagnostic yield for OSSN. There was also no improvement in results when analysing methylene blue according to different staining patterns (diffuse, focal or speckled).

This was the largest reported study of OSSN in South Africa. There were some limitations in the study. Patients that had incision biopsy may have had a histology result that was not representative of the grade of the lesion. For example, an area that was biopsied may have been reported as CIN3, whereas SCC may have been present in the lesion elsewhere in the mass. Histology was regarded as the gold standard in this study, with an assumed sensitivity and specificity of 100%. We acknowledge that, in reality, histology does not have perfect sensitivity and specificity. There were only a few patients with a diagnosis of CiS, which limited the analysis of this subgroup. There were however a large number in the CIN and SCC subgroups that were representative. A large number of patients had shadowing of the maximum epithelial thickness on OCT which had an effect on analysis of the ROC curve. We only included patients with OSSN in this study and therefore cannot comment on the ability of OCT to distinguish OSSN from other conjunctival malignancies. Twenty-seven per cent of cytology patients were excluded from analysis as their specimens did not have adequate cellularity for analysis. Details of the liquid-based medium are not available as this is proprietary information. The strength of the study is that it had a large number of patients with OSSN that had three non-invasive diagnostic modalities compared to the gold standard, histology.

Our study showed that OCT is the most reliable non-invasive diagnostic modality for OSSN. We are the first to report the use of liquid-based cytology in the diagnosis of OSSN. Cytology performed well, although it did not yield results that are comparable to previously reported traditional cytological assessments. Future studies could investigate this further.

## Summary

### What is known about this topic

- Ocular surface squamous neoplasia is the most common ocular surface tumour.
- Histology is the gold standard for diagnosis.
- OCT, cytology and methylene blue have been described for the diagnosis of OSSN.

### What this study adds

- OCT yielded better results than cytology or methylene blue stain for the diagnosis of OSSN.
- The epithelial thickness cutoff used in this study for the diagnosis of OSSN on OCT was 140 um.
- Methylene blue had a high sensitivity, but low specificity, making it a good screening test.
- Liquid-based cytology did not yield results comparable to traditional cytology for OSSN.
- Leukoplakia had a negative effect on the yield of cytology.

### How this study might affect research, practice or policy

- OCT can be used for the diagnosis of OSSN, allowing for outpatient management with topical chemo or immunotherapy.
- Studies can evaluate ways to improve yield on liquid-based cytology, as this still holds benefits over traditional cytology, such as the ability to perform PCR for HPV.
- Cytology may be a good option in resource-constrained settings where an OCT is not affordable, or theatre access is limited.
- Methylene blue can be used in primary care settings to assess the urgency of referral for conjunctival masses.

### Supplementary information

Supplement 1

Supplement 2

Supplement 3

Supplement 4

Supplement 5

## Data Availability

Data are available from the corresponding author on request.
