# Supplementary material for: Comparison of non-invasive diagnostic modalities for ocular surface squamous neoplasia at a tertiary hospital, South Africa
Source: Eye (Lond). 2023 Nov 23;38(6):1118–24. doi: 10.1038/s41433-023-02833-0 (PMC11009401; doi:10.1038/s41433-023-02833-0)
Supplement: Supplementary file 1 — Supplement 1 [file 41433_2023_2833_MOESM1_ESM.docx]

# Supplement 1

Supplement 1: American Joint Committee on Cancer staging for ocular surface squamous neoplasia.

| **T Category** | **T Criteria** |
| --- | --- |
| TX | Primary tumour cannot be assessed |
| T0 | No evidence of primary tumour |
| Tis | Carcinoma *in situ* |
| T1 | Tumour (≤5 mm in greatest dimension) invades through the conjunctival basement membrane without invasion of adjacent structures |
| T2 | Tumour (>5 mm in greatest dimension) invades through the conjunctival basement membrane without invasion of adjacent structures |
| T3 | Tumour invades adjacent structures (excluding the orbit) |
| T4 | Tumour invades the orbit with or without further extension |
| T4a | Tumour invades orbital soft tissues without bone invasion |
| T4b | Tumour invades bone |
| T4c | Tumour invades adjacent paranasal sinuses |
| T4d | Tumour invades brain |

Source: Amin MB, Edge SB, Greene FL, et al., editors. AJCC cancer staging manual. Cham: Springer International Publishing; 2017 [cited 2023 October 23]. Available from https://www.springer.com/gp/book/9783319406176
