# Supplementary material for: Comparison of non-invasive diagnostic modalities for ocular surface squamous neoplasia at a tertiary hospital, South Africa
Source: Eye (Lond). 2023 Nov 23;38(6):1118–24. doi: 10.1038/s41433-023-02833-0 (PMC11009401; doi:10.1038/s41433-023-02833-0)
Supplement: Supplementary file 2 — Supplement 2 [file 41433_2023_2833_MOESM2_ESM.docx]

**Supplement 2:** Baseline characteristics and histology of cases and controls.

|  | **OSSN (n=130, patients, n=135 lesions)** | **Benign (n=45 patients, n=47 lesions)** | **p-value** |
| --- | --- | --- | --- |
|  |  |  |  |
| **Baseline Characteristics (n=175)** | | | |
| **Median age in years (IQR)** | 44 (35-51) | 49 (40-56) | 0.02 |
| **Sex (%)** |  |  | 0.53 |
| **Male** | 62 (48) | 19 (42) |  |
| **Female** | 68 (52) | 26 (58) |  |
| **Race (%)** |  |  | 0.30 |
| **Black African** | 127 (98) | 45 (100) |  |
| **Mixed Race** | 3 (2) | 0 |  |
| **HIV positive (%)** | 93 (74)  (n=126) | 14 (32)  (n=42) | <0.001 |
| **Median CD4 at presentation, cells/uL (IQR)** | 202 (120-415) | 595 (270-722) | 0.003 |
| **Median VL at presentation, log copies/ml (IQR)** | 2.86 (1-4.76) | 1 (1-2.02) | 0.004 |
| **Morphology (%)** |  |  |  |
| **Fibrovascular** | 15 (12) | 31 (69) | <0.001 |
| **Nodular** | 4 (3) | 2 (4) | 0.65 |
| **Diffuse** | 2 (2) | 0 | 1.00 |
| **Placoid** | 110 (85) | 12 (27) | <0.001 |
| **Leukoplakic** | 67 (61) | 6 (50) | <0.001 |
| **Gelatinous** | 45 (41) | 5 (42) | 0.003 |
| **Papilliform** | 14 (13) | 2 (17) | 0.25 |
| **Median surface area, mm^2^ (IQR)** | 22 (9-33.4) | 17.5 (12.25-22) | 0.01 |
|  |  |  |  |
| **Histology (n=182)** | | | |
| **Benign (%)** |  | 47 (26) |  |
| **Pterygium** |  | 45 (96) |  |
| **Papilloma** |  | 1 (2) |  |
| **Naevus** |  | 1 (2) |  |
| **OSSN (%)** | 135 (74) |  |  |
| **CIN** | 112 (83) |  |  |
| **CIN 1** | 36 (32) |  |  |
| **CIN2** | 35 (31) |  |  |
| **CIN 3** | 41 (37) |  |  |
| **CiS** |  |  |  |
| **SCC** | 17 (13) |  |  |

OSSN: ocular surface squamous neoplasia

CIN: conjunctival intra-epithelial neoplasia

CiS: squamous cell carcinoma in-situ

SCC: Squamous cell carcinoma

IQR: Interquartile range

VL: Viral load
