# Supplementary material for: Comparison of non-invasive diagnostic modalities for ocular surface squamous neoplasia at a tertiary hospital, South Africa
Source: Eye (Lond). 2023 Nov 23;38(6):1118–24. doi: 10.1038/s41433-023-02833-0 (PMC11009401; doi:10.1038/s41433-023-02833-0)
Supplement: Supplementary file 3 — Supplement 3 [file 41433_2023_2833_MOESM3_ESM.docx]

**Supplement 3:** Comparison of the diagnostic utility of cytology, OCT, methylene stain and a combination of tests for the diagnosis of OSSN when compared to a histological diagnosis.

|  | **Sensitivity** | **Specificity** | **PPV** | **NPV** | **+ Likelihood Ratio** | **- Likelihood Ratio** |
| --- | --- | --- | --- | --- | --- | --- |
| **Cytology** | 72.4 (62.5 – 81.0) | 74.3 (56.7 – 87.5) | 88.8 (79.7 – 94.7) | 49.1 (35.1 – 63.2) | 2.82 (1.58 – 5.01) | 0.37 (0.25 – 0.54) |
| Cytology in OSSN with leukoplakia | 73.5 (58.9 – 85.1) | 33.3 (4.3 – 77.7) | 90.0 (76.3 – 97.2) | 13.3 (1.7 – 40.5) | 1.10 (0.61 – 1.99) | 0.80 (0.23 – 2.71) |
| Cytology in OSSN without leukoplakia | 71.4 (56.7 – 83.4) | 82.8 (64.2 – 94.2) | 87.5 (73.2 – 95.8) | 63.2 (46.0 – 78.2) | 4.14 (1.83 – 9.38) | 0.35 (0.22 – 0.55) |
| **OCT** | 87.2 (80.0 – 92.5) | 75.6 (60.5 – 87.1) | 90.8 (84.2 – 95.3) | 68.0 (53.3 – 80.5) | 3.57 (2.12 – 5.99) | 0.17 (0.10 – 0.28) |
| **Methylene** | 91.9 (85.9 – 95.9) | 55.3 (40.1 – 69.8) | 85.5 (78.7 – 90.8) | 70.3 (53.0 – 84.1) | 2.06 (1.49 – 2.84) | 0.15 (0.08 – 0.27) |
| **OCT or Cytology*** | 90.3 (84.0 – 94.7) | 66.0 (50.7 – 79.1) | 88.3 (81.7 – 93.2) | 70.5 (54.8 – 83.2) | 2.65 (1.77 – 3.96) | 0.15 (0.08 – 0.26) |
| **OCT or leukoplakia^#^** | 84.4 (77.2 – 90.1) | 72.3 (57.4 – 84.4) | 89.8 (83.1 – 94.4) | 61.8 (47.7 – 74.6) | 3.05 (1.91 – 4.87) | 0.22 (0.14 – 0.33) |
| **OCT or Methylene**** | 92.6 (86.8 – 96.4) | 48.9 (34.1 – 63.9) | 83.9 (77.0 – 89.4) | 69.7 (51.3 – 84.4) | 1.81 (1.37 – 2.41) | 0.15 (0.08 – 0.29) |
| **Leukoplakia or cytology^##^** | 78.5 (70.6 – 85.1) | 76.6 (62.0 – 87.7) | 90.6 (83.8 – 95.2) | 55.4 (42.5 – 67.7) | 3.35 (1.99 – 5.67) | 0.28 (0.20 – 0.40) |
| **Methylene or cytology***** | 94.8 (89.6 – 97.9) | 46.8 (32.1 – 61.9) | 83.7 (76.8 – 89.1) | 75.9 (56.5 – 89.7) | 1.78 (1.36 – 2.34) | 0.11 (0.05 – 0.24) |
| **Leukoplakia and OSSN on OCT of non-leukoplakic masses^###^** | 88.4 (81.5 – 93.3) | 71.1 (55.7 – 83.6) | 89.8 (83.1 – 94.4) | 68.1 (52.9 – 80.9) | 3.06 (1.93 – 4.86) | 0.16 (0.10 – 0.27) |
| **Leukoplakia and OSSN on cytology of non-leukoplakic masses****** | 88.3 (81.2 – 93.5) | 68.6 (50.7 – 83.1) | 90.6 (83.8 – 95.2) | 63.2 (46.0 – 78.2) | 2.81 (1.72 – 4.60) | 0.17 (0.10 – 0.29) |
| **Methylene staining and OSSN on OCT of staining masses^####^** | 94.7 (88.9 – 98.0) | 60.0 (36.1 – 80.9) | 93.1 (86.9 – 97.0) | 66.7 (41.0 – 86.7) | 2.37 (1.38 – 4.06) | 0.09 (0.04 – 0.21) |
| **Methylene staining and OSSN on cytology of staining masses******* | 73.6 (63.3 – 82.3) | 70.6 (44.0 – 89.7) | 93.1 (84.5 – 97.7) | 33.3 (18.6 – 51.0) | 2.50 (1.19 – 5.28) | 0.37 (0.24 – 0.59) |
| **OSSN on OCT and cytology of positive masses on OCT^#####^** | 73.8 (62.7 – 83.0) | 50.0 (15.7 – 84.3) | 93.7 (84.5 – 98.2) | 16.0 (4.5 – 36.1) | 1.48 (0.73 – 2.99) | 0.53 (0.24 – 1.15) |

OSSN: ocular surface squamous neoplasia

OCT: optical coherence tomography

PPV: positive predictive value

NPV: negative predicative value

* Mass was considered to be OSSN if it was positive for OSSN on OCT criteria **OR** on cytology.

# Mass was considered to be OSSN if it was positive for OSSN on OCT criteria **OR** had leukoplakia clinically.

** Mass was considered to be OSSN if it was positive for OSSN on OCT criteria **OR** stained with methylene blue.

## Mass was considered to be OSSN if it had leukoplakia **OR** had features of OSSN on cytology.

*** Mass was considered to be OSSN if it had leukoplakia **OR** had features of OSSN on cytology.

### Mass was considered to be OSSN if it had leukoplakia **AND** if the remaining non-leukoplakic masses had features of OSSN on OCT. Leukoplakia was used as a screening test before OCT.

**** Mass was considered to be OSSN if it had leukoplakia **AND** if the remaining non-leukoplakic masses had features of OSSN on cytology. Leukoplakia was used as a screening test before OCT.

#### Mass was considered to be OSSN if it stained with methylene blue **AND** if the staining masses has features of OSSN on OCT. The methylene blue was used as a screening test before OCT.

***** Mass was considered to be OSSN if it stained with methylene blue **AND** if the staining masses has features of OSSN on cytology. The methylene blue was used as a screening test before OCT.

##### Mass was considered to be OSSN if it had features of OSSN on OCT **AND** if those masses has features of OSSN on cytology. The OCT was used as a screening test before cytology.
