# Supplementary material for: Comparison of non-invasive diagnostic modalities for ocular surface squamous neoplasia at a tertiary hospital, South Africa
Source: Eye (Lond). 2023 Nov 23;38(6):1118–24. doi: 10.1038/s41433-023-02833-0 (PMC11009401; doi:10.1038/s41433-023-02833-0)
Supplement: Supplementary file 4 — Supplement 4 [file 41433_2023_2833_MOESM4_ESM.docx]

**Supplement 4**: Receiver operator curve of maximum epithelial thickness measurements on optical coherence tomography. An epithelial thickness cut-off of 140um used for the diagnosis of OSSN, which correctly classified ocular surface squamous neoplasia in 77% of cases.


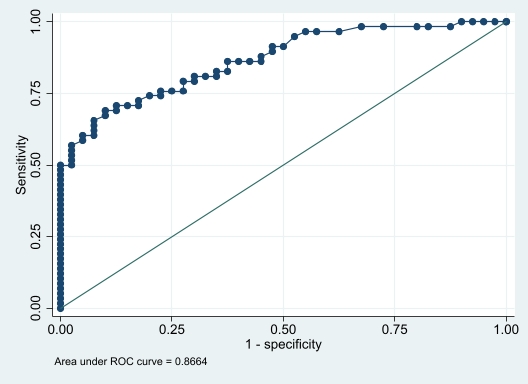


Thicker Epithelium

Epithelium not measured

Thinner Epithelium
