# Supplementary material for: Comparison of non-invasive diagnostic modalities for ocular surface squamous neoplasia at a tertiary hospital, South Africa
Source: Eye (Lond). 2023 Nov 23;38(6):1118–24. doi: 10.1038/s41433-023-02833-0 (PMC11009401; doi:10.1038/s41433-023-02833-0)
Supplement: Supplementary file 5 — Supplement 5 [file 41433_2023_2833_MOESM5_ESM.docx]

**Supplement 5:** Receiver operating characteristic curve for the detection of ocular surface squamous neoplasia on (A) optical coherence tomography, (B) impression cytology, and (C) methylene blue stain.


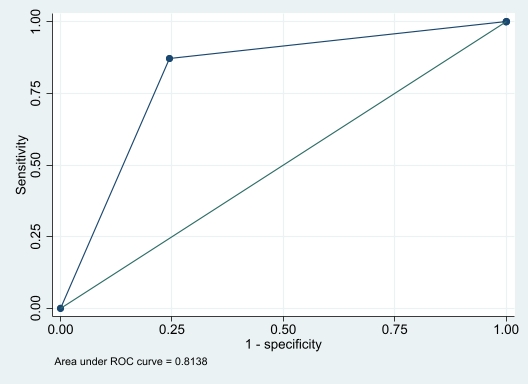

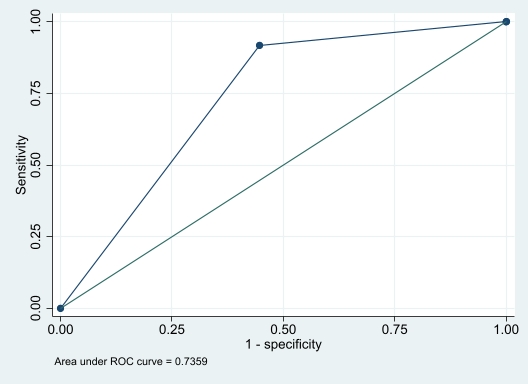

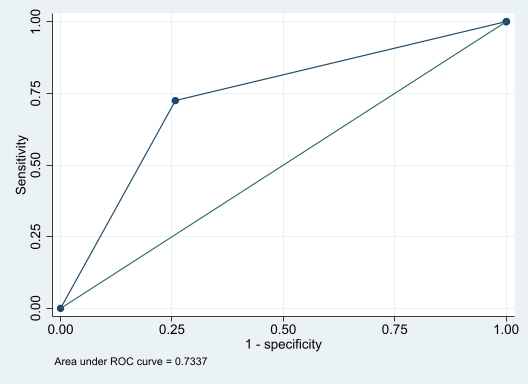


**A**

**C**

**B**
